# FAT1 and PTPN14 Regulate the Malignant Progression and Chemotherapy Resistance of Esophageal Cancer through the Hippo Signaling Pathway

**DOI:** 10.1155/2021/9290372

**Published:** 2021-10-19

**Authors:** Yingzhi Lu, Zhenxin Wang, Ling Zhou, Zhaoming Ma, Jianguo Zhang, Yan Wu, Yan Shao, Yunyun Yang

**Affiliations:** ^1^Department of Oncology, The First Affiliated Hospital of Soochow University, Suzhou, Jiangsu 215006, China; ^2^Department of Oncology, The Second People's Hospital of Lianyungang (The Oncology Hospital of Lianyungang), No 161, XinFu Road, Lianyungang, Jiangsu 222000, China; ^3^Department of Nephrology, The Second's Hospital of Lianyungang (The Oncology Hospital of Lianyungang), No 161, XinFu Road, Lianyungang, Jiangsu 222000, China; ^4^Department of Medical Oncology, The Second People's Hospital of Lianyungang (The Oncology Hospital of Lianyungang), No 161, XinFu Road, Lianyungang, Jiangsu 222000, China

## Abstract

**Background:**

Esophageal cancer (EC) is a common malignant tumor, which brings heavy economic burden to patients and society. Therefore, it is important to understand the molecular mechanism of recurrence, metastasis, and drug resistance of esophageal cancer.

**Methods:**

Human esophageal cancer cell line TE13 (poorly differentiated squamous cell carcinoma) and normal human esophageal epithelial cell line het-1a were selected for aseptic culture. At the same time, 6 bottles of TE13 cell line were inoculated in logarithmic phase. Cell apoptosis was analyzed by flow cytometry (FCM). Cell clone formation assay was used to analyze the proliferation. Fibronectin-coated dishes were used to detect the characteristics of cell adhesion to extracellular matrix. The Transwell method was used to detect the cell invasion ability. Western blot was used to analyze the expression of Yap1, PTPN14, FAT1, and Myc.

**Results:**

Results showed that FAT1 and PTPN14 were downregulated, while Yap1 was upregulated in esophageal cancer tissues. FAT1 inhibited the proliferation, adhesion, and invasion of human esophageal cancer cell lines, which might be associated with the upregulation of PTPN14 and the inhibition of Yap1 and Myc.

**Conclusion:**

The results suggested that PTPN14 and FAT1 could regulate malignant progression and chemotherapy resistance of esophageal cancer based on the Hippo signaling pathway.

## 1. Introduction

Esophageal cancer (EC) is a common malignant tumor of the digestive tract, which brings heavy economic burden to patients and society [1]. Due to its complicated pathogenesis, difficult prevention, and lack of early diagnosis, the morbidity and mortality rates of EC are still high [2–4].

The Hippo pathway is first found in the process of functional genetic screening in Drosophila, which is highly conservative and plays a role in regulating organ size and maintaining the dynamic balance of cell proliferation or apoptosis [5–7]. Yes-associated protein (Yap) is an important target of the Hippo signaling pathway. Under normal circumstances, the activity of the Hippo-Yap signaling pathway is strictly regulated, and once the pathway is out of balance, it will lead to the occurrence and development of a variety of diseases and even malignant tumors [8–10]. Yap1, a major effector and regulatory protein in the classical Hippo-YAP signaling pathway, regulates the expression of several target genes by entering into the nucleus as a transcriptional coactivated form. Its expression is significantly increased in various tumor tissues and cells [10–12]. It has been found that increased expression of Yap1 is associated with the development, progression, and drug resistance of esophageal cancer.

Protein tyrosine phosphatase nonreceptor type 14 (PTPN14) is a member of the protein tyrosine phosphatase (PTPs) family encoded by 14 genes. PTPs can be involved in the regulation of a variety of cell processes, including cell proliferation, differentiation, mitosis, and even malignant transformation [13–15]. Fat cadherins are very large molecules, including FAT1, FAT2, FAT3, and FAT4. Among them, FAT1 is a transmembrane protein which plays an important role in the regulation of cell adhesion and growth, migration, actin dynamics and orientation in tumor progression [16–18]. However, how FAT1 and PTPN14 regulate the malignant progression and chemotherapy resistance of esophageal cancer is still unclear.

Here, we studied the role of FAT1 and PTPN14 in the regulation of cell proliferation, adhesion, and invasion during esophageal cancer progression and the possible association with the Hippo pathway. This study might provide a theoretical basis on the roles of FAT1 and PTPN14 in the occurrence and development of esophageal cancer.

## 2. Material and Methods

### 2.1. Tissue Collection

The specimens of 20 cases of esophageal cancer adjacent tissues and 20 cases of esophageal cancer patients were collected from EC patients who came to the The Second People's Hospital of Lianyungang (The Oncology Hospital of Lianyungang). All patients received no chemotherapy or radiotherapy before. Tissues were collected immediately after resection and were stored at -80°C. This study was approved by the Ethical Committee of The Second People's Hospital of Lianyungang (The Oncology Hospital of Lianyungang).

### 2.2. RNA Extraction and qRT-PCR

Total RNA was extracted using TRIzol (Invitrogen, Grand Island, NY) according to the manufacturer's instruction. The mRNA expression levels of Yap1, PTPN14, and FAT1 were analyzed by qRT-PCR. The cDNA was synthesized using the PrimeScript RT Reagent Kit (TaKaRa Bio, Shiga, Japan) following the manufacturer's instructions. Real-time PCR was performed on a Roche Light Cycler 480 (Roche) using the SYBR Green PCR Master Mix (TaKaRa Bio, Shiga, Japan). Each measurement was performed in triplicate, and the results were normalized by the expression of the GADPH gene. Fold change relative to mean value was determined by 2^-*ΔΔ*Ct^ [19].

### 2.3. Immunohistochemistry (IHC)

For IHC, 10% neutral formalin was immobilized, dehydrated, paraffin-embedded, and cut into 4-micron-thick tissue sections. Samples were then stained with hematoxylin- and eosin- (*H&E*) staining, immersed with 3% H_2_O_2_, and incubated with primary antibodies against related proteins (anti-FAT1 ab198892 1/200, anti-Yap1 ab52771 1/50, Abcam). The second antibody (goat anti-rabbit IgG H&L (FITC) or goat anti-rabbit IgG H&L (Biotin), Abcam) was labeled with FITC or biotin and stained with the ABC method. The negative control group, positive control group, and fluorescent marker control group were set up. For quantification, the Allred scoring system was used [20]: the sum of staining intensity 0 (no staining), 1 (weak staining), 2 (moderate staining), and 3 (strong staining) and the percentage scores of the stained area 0 (none), 1 (<1/100), 2 (1/100 to 1/10), 3 (1/10 to 1/3), 4 (1/3 to 2/3), and 5 (>2/3) were regarded the final IHC score (0~2, negative; 3~4, weak; 5~6, moderate; and 7~8, strong).

### 2.4. Cell Culture and Transfection

All cell lines used in the present study was purchased from the Cell Bank of Type Culture Collection (Chinese Academy of Sciences, Shanghai, China). Three human esophageal cancer cell lines TE13 (poorly differentiated squamous cell carcinoma), KYSE140 (moderately differentiated squamous cell carcinoma), and SEG1 (well differentiated adenocarcinoma) were cultured strictly in accordance with aseptic culture. TE13 and normal human esophageal epithelial cell line het-1a were selected for aseptic culture. For cell transfection, 6 bottles of TE13 cell line were inoculated in logarithmic phase and transfected with FAT1 overexpressed plasmid (ov-FAT1) or FAT1 small interfering RNA plasmid (si-FAT1) using the lipo6000 reagent (Beyotime Biotechnology, China) [21]. The ov-FAT1 and si-FAT1 were all synthesized and purchased from Applied Biological Materials (GenePharma, Shanghai, China). For the treatment of cisplatin, cisplatin (5 *μ*g/ml) was administered at the same time.

### 2.5. Flow Cytometry (FCM)

FCM analysis for cell cycle and apoptosis was conducted 72 hours posttransfection, using the nuclear stain DAPI (40,6-diamidino-2-phenylindole) or Annexin V-FITC/7-AAD Kit. Then, the apoptosis characteristics of each group were analyzed using a FSCAN flow cytometer (BD Biosciences, USA) [22].

### 2.6. Cell Clone Formation Assay, Transwell Assay, and Adhesion Assay

Cell viability was determined by clone formation assay. Briefly, after 72 hours of transfection, cells with density of 4 × 10^3^ were seeded into 6-well dishes, incubating for 14 days, then fixed with glutaraldehyde, and stained with crystal violet. The positive colony formation was manually counted under a light microscope (Zeiss, Germany).

The Transwell method was used to detect the invasion ability of cells in each experimental group. Briefly, 3 × 10^4^/ml cells were seeded onto the upper chamber. The cells were then cultured in serum-free media, were incubated for 24 h, and were then fixed and stained using Crystal Violet. Fibronectin-coated dishes were used to detect the characteristics of cell adhesion to extracellular matrix in each experimental group as reported elsewhere [23].

### 2.7. Western Blotting

Western blotting was used to evaluate the protein expression of Yap1, PTPN14, FAT1, and Myc in each cell line. Samples were subjected to SDS-PAGE, transferred to polyvinylidene difluoride membranes and then blocked by 5% nonfat milk at room temperature for 1 h. Then, membranes were incubated with primary antibodies of anti-Yap1 ab52771 1/5000, anti-PTPN14 ab204321 1/2000, anti-FAT1 ab190242 1/2000, and anti-Myc ab9106 1/2000 (Abcam) at 4°C overnight, following with incubation with a goat anti-rabbit secondary antibody (ab205718, 1/2000, Abcam) at 37°C for 45 min. The bound antibodies were detected using the enhanced chemiluminescence plus Western blotting detection system (Bio-Rad) [24]. Relative protein expression was quantified using the Image-Pro Plus software (version 6.0; Media Cybernetics, Inc.).

### 2.8. Statistical Analysis

All quantified data represent an average of at least triplicate samples or as indicated. Error bars represent the SD of the mean. Statistical significance was determined by the Student's *t* test, and 2-tailed *P* values less than 0.05 were considered significant.

## 3. Result

### 3.1. Yap1 Is Upregulated while PTPN14 and FAT1 Are Downregulated in Esophageal Cancer Tissues

**Figure 1:**
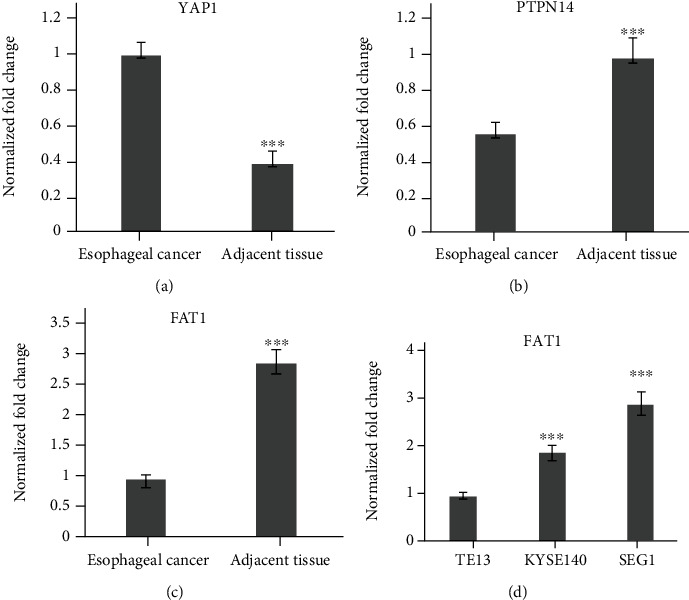
The mRNA expression of Yap1 PRPN14 and FAT1 in esophageal cancer. (a) Yap1 mRNA was highly expressed in the tissues of esophageal carcinoma (*n* = 20), but low in esophageal carcinoma adjacent tissues (*n* = 20). The expression of PTPN14 (b) and FAT1 (c) was low in the tissues of esophageal carcinoma, but high in the adjacent tissues of esophageal carcinoma. (d) FAT1 was highly expressed in the SEG1, but low in TE13. ^∗∗∗^*P* < 0.001 vs. the EC tissue. ^###^*P* < 0.001 vs. TE13.

The specimens of 20 cases of esophageal cancer adjacent tissues and 20 cases of esophageal cancer patients were collected. The mRNA levels of Yap1, PTPN14 and FAT1 were analyzed by qPCR. The results showed that Yap1 mRNA was highly expressed in esophageal carcinoma tissues, but low in the adjacent tissues of esophageal carcinoma (Figure 1(a)). The expression levels of PTPN14 and FAT1 were low in the tissues of esophageal carcinoma, but high in the adjacent tissues of esophageal carcinoma (Figures 1(b) and 1(c)).

**Figure 2:**
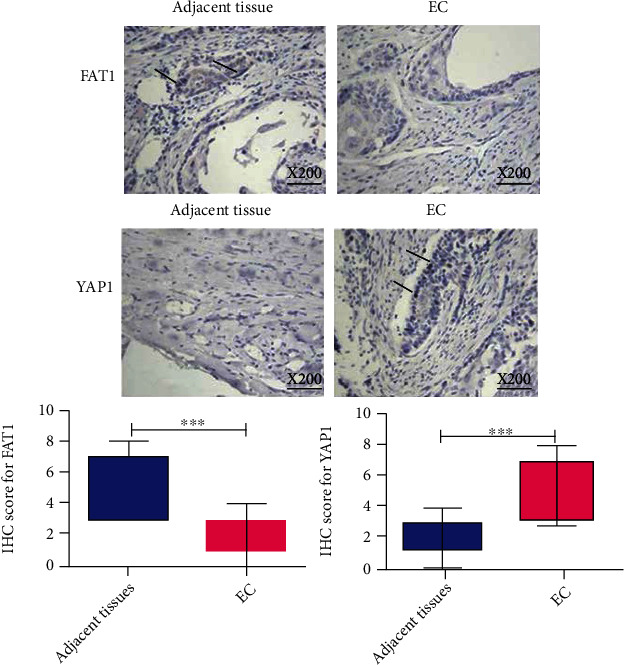
The protein expression of Yap1 and FAT1 in esophageal cancer tissues. FAT1 protein was highly expressed in the adjacent tissues of esophageal carcinoma, but low in esophageal carcinoma tissues. The expression of Yap1 was low in the adjacent tissues of esophageal carcinoma, but high in the tissues of esophageal carcinoma (magnification: 200 X). ^∗∗∗^*P* < 0.001.

Besides, immunohistochemistry was conducted on the same samples of esophageal cancer to analyze the relative protein expression of FAT1 and Yap1. The results showed that FAT1 protein was highly expressed in the adjacent tissues of esophageal carcinoma, but low in esophageal carcinoma tissues. The expression of Yap1 was low in the adjacent tissues of esophageal carcinoma, but high in the tissues of esophageal carcinoma (Figure 2).

### 3.2. FAT1 Was Highly Expressed in the Well Differentiated Adenocarcinoma

Three human esophageal cancer cell lines TE13, KYSE140, and SEG1 were cultured strictly in accordance with aseptic culture. qRT-PCR results showed that FAT1 was highly expressed in the SEG1, but low in TE13 (Figure 1(d)), suggesting FAT1 might inhibit the proliferation and progression of esophageal cancer.

### 3.3. FAT1 Inhibits the Proliferation and Adhesion of Human Esophageal Cancer Cell Lines

**Figure 3:**
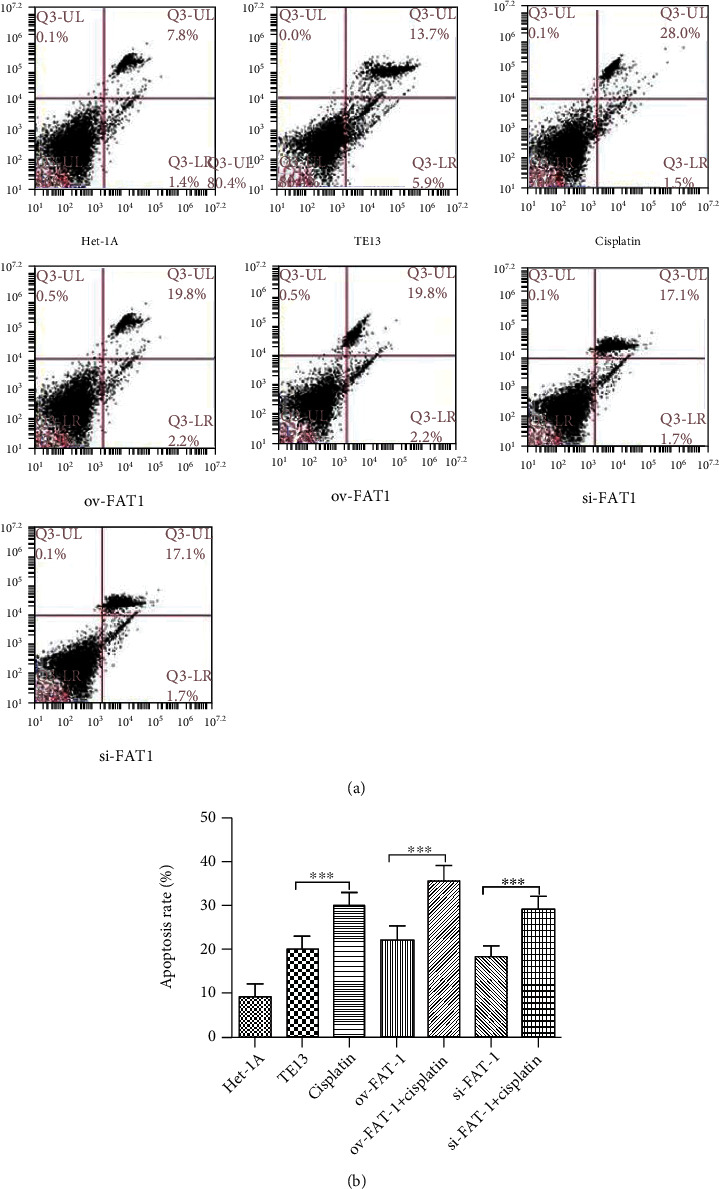
FAT1 promotes the apoptosis of human esophageal cancer cell lines. Human esophageal cancer cell lines het-1A and TE13 were cultured strictly. (a) FCM analysis for cell cycle and apoptosis was done 72 hours posttransfection, using the Annexin V-FITC/7-AAD Kit for apoptosis analysis. (b) Apoptosis rate of (a).

**Figure 4:**
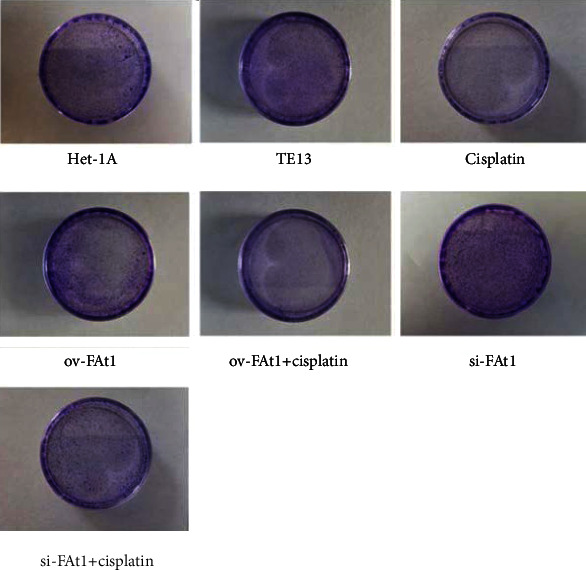
FAT1 inhibits the cell viability of human esophageal cancer cell lines. Clone formation assay was used to detect the proliferation ability of cells in each experimental group (het-1A cell line, TE13 cell line, TE13 cells treated with cisplatin, and TE13 cells transfected with ov-FAT1 and si-FAT or treated with cisplatin at the same time). The clones were fixed and stained using Crystal Violet.

**Figure 5:**
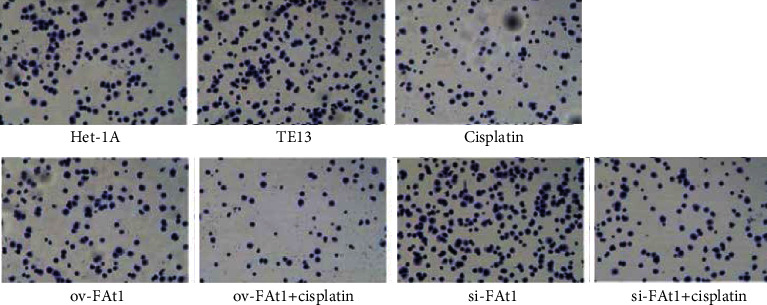
FAT1 inhibits the adhesion of human esophageal cancer cell lines. Fibronectin-coated dishes were used to detect the characteristics of cell adhesion to extracellular matrix in each experimental group (het-1A cell line, TE13 cell line, TE13 cells treated with cisplatin, and TE13 cells transfected with ov-FAT1 and si-FAT or treated with cisplatin at the same time). Magnification: 200 X.

TE13 and the normal human esophageal epithelial cell line het-1a were selected for aseptic culture. At the same time, the TE13 cell line was inoculated in logarithmic phase, which was numbered and transfected with FAT1 and si-FAT1. Additionally, cisplatin was given to inhibit the cell development. Then, the apoptosis characteristics of each group were analyzed by flow cytometry (FCM). The results demonstrated that FAT1 inhibited the proliferation of human esophageal cancer cell lines (Figure 3). Cell clone formation assay also demonstrated that the FAT1 inhibited the cell viability of human esophageal cancer cell lines (Figure 4). Cell adhesion assay also demonstrated that the FAT1 inhibited the cell adhesion of human esophageal cancer cell lines (Figure 5).

### 3.4. FAT1 Inhibits the Invasion of Human Esophageal cancer Cell Lines

**Figure 6:**
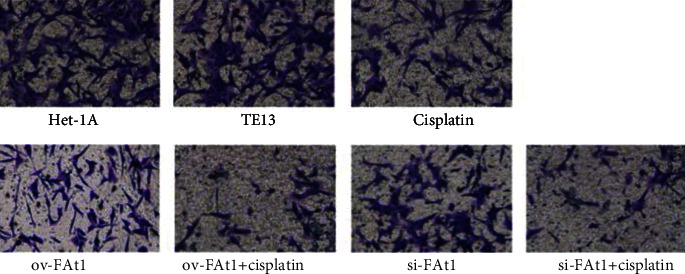
FAT1 inhibit the invasion of human esophageal cancer cell lines. Magnification: 200 X. The Transwell method was used to detect the invasion ability of cells in each experimental group (het-1A cell line, TE13 cell line, TE13 cells treated with cisplatin, and TE13 cells transfected with ov-FAT1 and si-FAT or treated with cisplatin at the same time). The activation of FAT1 inhibits the invasion of human esophageal cancer cell lines. Magnification: 200 X.

**Figure 7:**
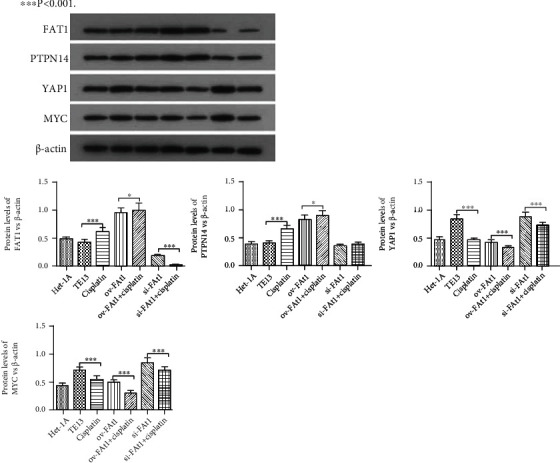
Western blot was performed to detect the protein levels of Yap1, PTPN14, FAT1, and Myc. WB was used to evaluate the expression of Yap1, PTPN14, FAT1, and Myc. FAT1 significantly enhanced the protein level of PTPN14 and inhibited the protein levels of Yap1 and Myc. Meanwhile, knockdown of FAT1 significantly downregulated the protein level of PTPN14 and increased the protein levels of Yap1 and Myc. ^∗^*P* < 0.05, ^∗∗∗^*P* < 0.001.

To evaluate the role of FAT1 in the invasion of human esophageal cancer cell lines, Transwell assay was conducted. The result demonstrated that FAT1 inhibited the invasion of human esophageal cancer cell lines (Figure 6). Western blot was performed to detect the protein levels of Yap1, PTPN14, FAT1, and Myc. The results showed that FAT1 significantly enhanced the protein level of PTPN14 and inhibited the protein levels of Yap and Myc. Meanwhile, knockdown of FAT1 significantly downregulated the protein level of PTPN14 and increased the protein levels of Yap1 and Myc (Figure 7). Together, these data showed that FAT1 inhibited the cell proliferation, adhesion, and invasion of human esophageal cancer, which might be associated with the upregulation of PTPN14 and inhibition of Yap1 and Myc.

## 4. Discussion

In this study, we examined the expression levels of Yap1, PTPN14, and FAT1 in human esophageal cancer cell lines and tissues. We also studied FAT1/PTPN14 on proliferation, adhesion, and invasion characteristics of esophageal cancer cell lines by FACS, clone formation assay, and the Transwell method. Our results showed that FAT1 and PTPN14 are downregulated while Yap1 is upregulated in esophageal cancer tissues. FAT1 promotes the expression of PTPN14 and downregulates the expressions of Yap1 and Myc, to inhibit the proliferation, adhesion, and invasion of human esophageal cancer cell lines.

Notably, the invasion and metastasis of tumor cells are two of the important biological characteristics of malignant tumors. Abnormal expression of Yap1 can promote the malignant transformation of cells and induce the occurrence and development of tumors. The Yap gene is located in the long arm of chromosome 11, including Yap1 and Yap2 subtypes. Yap1, as a key effector molecule of the Hippo signaling pathway, can directly participate in cell proliferation, apoptosis, and other biological behaviors after phosphorylation [25–27]. In EC, it was also reported that Yap1 could mediate EGFR overexpression and confers chemoresistance [28].

In 1995, when scientists were looking for tyrosine phosphatase expressed in normal breast tissue, PTPN14 was cloned and found for the first time [29]. It is a member of the nonreceptor tyrosine phosphatase family. Studies have shown that PTPN14 has mutations in breast cancer, colon cancer, and other tumors [30–32]. Knockdown of PTPN14 can promote the invasion and metastasis of breast cancer cells [33]. In another study, it was found that PTPN14 was correlated with TNM stage and N stage in colorectal cancer [34]. In addition, PTPN14 has been considered to be involved in the regulation of cell proliferation.

FAT1 often functions as a tumor-suppressor or oncogene in different types of human cancer [16–18]. Some studies have shown the role of FAT1 in EC. It was found that FAT1 could suppress cell migration and invasion by affecting the cellular mechanical properties in esophageal squamous cell carcinoma [35]. In this study, we identified FAT1 as a tumor-suppressor which can inhibit the proliferation, adhesion, and invasion of human esophageal cancer cell lines. Through further analysis, we found that these proteins have regulatory network characteristics which have been proved in this study. Our data also implicated that FAT1 might function as the tumor suppression through the promotion of the expression of PTPN14 and inhibition of the expressions of Yap1 and Myc. Taken together, the results show that FAT1 plays an important role in the proliferation cycle of esophageal cancer, which suggest a theoretical basis for the roles of FAT1 and PTPN14 in the occurrence and development of esophageal cancer, and provide some new molecular targets for the treatment of esophageal cancer.

## Data Availability

The data used to support the findings of this study are available from the author upon request.
